# FDG-PET Positivity and Overall Survival in Renal Cell Carcinoma

**DOI:** 10.1001/jamanetworkopen.2022.42289

**Published:** 2022-11-16

**Authors:** Justin Ferdinandus, Ines Maríc, Christopher Darr, Claudia Kesch, Timo Bartel, Wolfgang Peter Fendler, Viktor Grünwald

**Affiliations:** 1 Department of Nuclear Medicine, University of Duisburg-Essen and German Cancer Consortium (DKTK)-University Hospital Essen, Essen, Germany; 2 Department of Urology, University Hospital Essen, University of Duisburg-Essen, Essen, Germany; 3 Department of Medical Oncology, Department of Medical Oncology, University Hospital Essen, University of Duisburg-Essen, Essen, Germany

## Abstract

This cohort study examines positron emission tomography in renal cell carcinoma and its association with overall survival among adults.

## Introduction

Positron emission tomography (PET) with 2-deoxy-2-[18F]fluoro-D-glucose (FDG) is not a standard modality in renal cell carcinoma (RCC) because of possible PET-negative disease.^1^ However, PET positivity could reflect more aggressive disease.^2^ We evaluated PET positivity in RCC and its association with overall survival.

## Methods

This cohort study followed the Strengthening the Reporting of Observational Studies in Epidemiology (STROBE) reporting guideline. The ethics committee of the University Hospital Essen approved the study and waived the requirement for consent given the retrospective nature of the study and use of deidentified patient data.

We retrospectively analyzed patients from the PET-KID database with RCC who received FDG-PET/computed tomography (CT) between 2010 and 2020. To be eligible for this study, patients needed to have at least 1 site of measurable disease by CT. PET positivity was rated analogue to Deauville criteria. Definitions and a patient flow chart are included in the Supplement.

The primary endpoint was overall survival (OS) measured from date of PET until death or last contact (censored). Kaplan-Meier methods, log-rank testing, and multivariable Cox regression were used to assess survival or prediction, respectively. Kendall rank test was performed to correlate PET avidity and histologic grading. *P* < .05 was regarded as statistically significant. Statistical analysis was performed using R version 3.4.1 (R Project for Statistical Computing). Statistical analysis was performed in July 2020.

## Results

This study included 90 patients between age 34 and 83 years, and 64 patients (71.1%) had metastatic RCC, and 56 patients (62.2%) had clear cell RCC. Cohort characteristics are given in the Table. The median (range) follow-up was 27.7 (0.5-122.0) months. All patients had uptake above background, and 56 patients (62.2%) were FDG-PET positive, 21 patients (23.3%) had high avidity, and 35 patients (38.9%) had intense avidity (ie, markedly above liver). PET positivity rates were similar in metastatic disease (47 [61.0%]) and nonmetastatic disease (9 [69.2%]). We found significant correlation between PET avidity and histologic grade (Kendall tau, 0.22; *P* = .03).

**Table.** Patient Characteristics

Characteristic	No. (%)		
	Initial diagnosis (n = 13)	Recurrent or metastatic (n = 77)	Overall (n = 90)
Age, median (range), y	66.0 (46.0-79.0)	64.0 (34.0-83.0)	64.0 (34.0-83.0)
Sex			
Female	3 (23.1)	23 (29.9)	26 (28.9)
Male	10 (76.9)	54 (70.1)	64 (71.1)
Months since initial diagnosis, median (range)	1.0 (0-3.0)	33.0 (1.0-436.0)	24.5 (0- 436.0)
Histology			
Clear-cell	7 (53.8)	49 (63.6)	56 (62.2)
Chromophobe	0	1 (1.3)	1 (1.1)
Papillary	0	3 (3.9)	3 (3.3)
Sarcomatoid	0	3 (3.9)	3 (3.3)
Not reported	6 (46.2)	21 (27.3)	27 (30.0)
Grade			
1	0	2 (2.6)	2 (2.2)
2	5 (38.5)	41 (53.2)	46 (51.1)
3	2 (15.4)	11 (14.3)	13 (14.4)
4	0 (0)	2 (2.6)	2 (2.2)
Not reported	6 (46.2)	21 (27.3)	27 (30.0)
T stage			
1	4 (30.8)	26 (33.8)	30 (33.3)
2	0 (0)	10 (13.0)	10 (11.1)
3	7 (53.8)	21 (27.3)	28 (31.1)
4	2 (15.4)	3 (3.9)	5 (5.6)
Not reported	0	17 (22.1)	17 (18.9)
Local disease (primary or recurrence)	10 (76.9)	26 (33.8)	36 (40.0)
Nodal metastases	4 (30.8)	46 (59.7)	50 (55.6)
Distant metastases	9 (69.2)	55 (71.4)	64 (71.1)
Previous tumor nephrectomy	0	72 (93.5)	72 (80.0)
Previous systemic therapy	0	22 (28.6)	22 (24.4)
PET positivity			
Negative	4 (30.8)	30 (39.0)	34 (37.8)
Positive	9 (69.2)	47 (61.0)	56 (62.2)

Abbreviation: PET, positron emission tomography.

Histologic grading, history of previous nephrectomy, presence of local disease (ie, primary or local recurrence), nodal involvement, as well as general PET positivity and quantitative measurements were associated with being significant estimators of OS in univariate Cox regression analyses. Of these factors, together with known estimators of survival, only PET positivity and previous nephrectomy were associated with significance in multivariable Cox regression (Figure).

**Figure.**
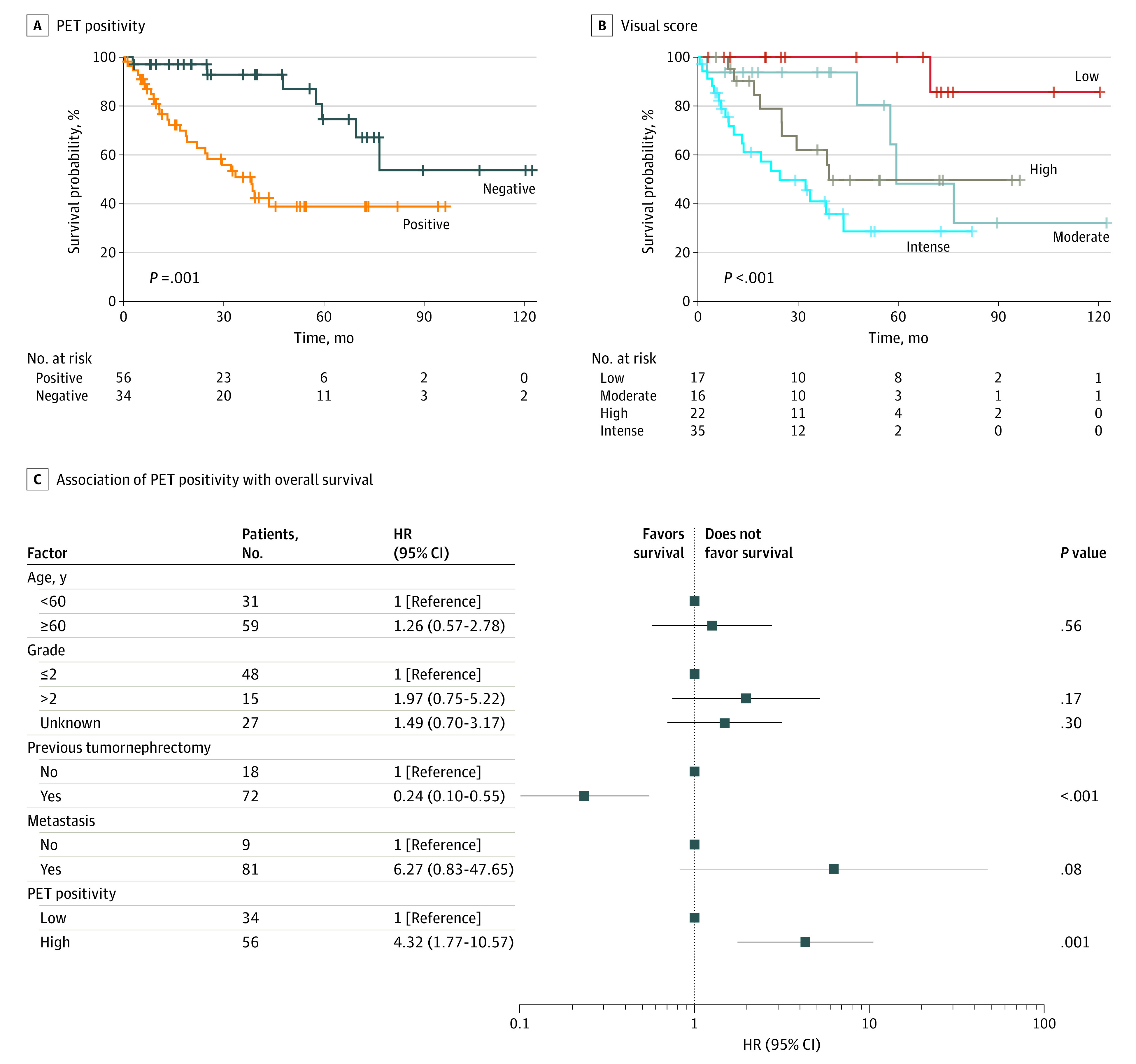
PET Positivity and Overall Survival HR, hazard ratio; PET, positron emission tomography.

PET avidity was significantly associated with OS (HR, 3.3; 95% CI, 1.5-7.4; *P* < .01). PET-negative patients had significantly longer survival (median not reached; median [IQR] follow-up, 47.5 [20.2-71.0] months) as compared with PET-positive patients (33.5 months; median [IQR] follow-up, 22.0 [8.8-39.6] months; *P* < *.*01) (Figure).

## Discussion

Our study suggested that most RCC are metabolically active (63.3% with high or intense uptake). Previous studies reported low accuracy for FDG-PET in RCC but indicated semiquantitative PET parameters to be associated with survival.^3,4,5,6^ We found an association between PET avidity and histologic grading, which suggested that PET positivity was associated with more aggressive disease and poor prognosis. Our study indicated that PET metabolism informs on prognosis in RCC, which renders PET imaging a putative marker to guide treatment decisions in RCC.

This study had limitations, including its retrospective nature, which is prone to selection bias. Our observational period is subject to run-time bias; although, we have not detected an association between time since diagnosis and OS.

## Conclusions

Most patients with RCC were FDG-PET positive, which was associated with aggressive disease and poor prognosis. Our data warrants further study to investigate FDG-PET imaging as a novel marker for prognosis, response, and treatment guidance of contemporary therapies in advanced or metastatic RCC.

## Notes

Supplement. **eTable.** Definitions of PET Positivity **eFigure.** Flow Chart
