# Supplementary material for: FDG-PET Positivity and Overall Survival in Renal Cell Carcinoma
Source: JAMA Netw Open. 2022 Nov 16;5(11):e2242289. doi: 10.1001/jamanetworkopen.2022.42289 (PMC9669811; doi:10.1001/jamanetworkopen.2022.42289)
Supplement: Supplement. — eTable. Definitions of PET Positivity eFigure. Flow Chart [file jamanetwopen-e2242289-s001.pdf]

Supplemental Online Content

Ferdinandus J, Maríc I, Darr C, et al. FDG-PET positivity and overall survival in renal cell carcinoma. *JAMA Netw Open*. 2022;5(11):e2242289. doi:10.1001/jamanetworkopen.2022.42289

eTable. Definitions of PET Positivity
eFigure. Flow Chart

This supplemental material has been provided by the authors to give readers additional information about their work.

© 2022 Ferdinandus J, et al. *JAMA Network Open.*

eTable. Definitions of PET Positivity

Score	label	Description
**1**	none	No uptake above the background (no uptake)
**2**	low	Uptake ≤ mediastinum (low uptake)
**3**	moderate	Uptake > mediastinum but ≤ liver (intermediate uptake)
**4**	high	Uptake moderately increased compared to the liver at any site
**5**	intense	Uptake markedly increased compared to the liver at any site

To determine visual rate of PET-positivity, images were read by a nuclear physician and evaluated using a five-point scoring system analogue to the *Deauville Criteria (1)*:

For the hottest lesion SUVmax, SUVpeak and SUVmean were recorded. Scores greater or equal to 4 and SUVpeak > SUVpeak Liver + 2 SD SUVmean of Liver were considered as PET-positive

© 2022 Ferdinandus J, et al. *JAMA Network Open.*

eFigure. Flow Chart

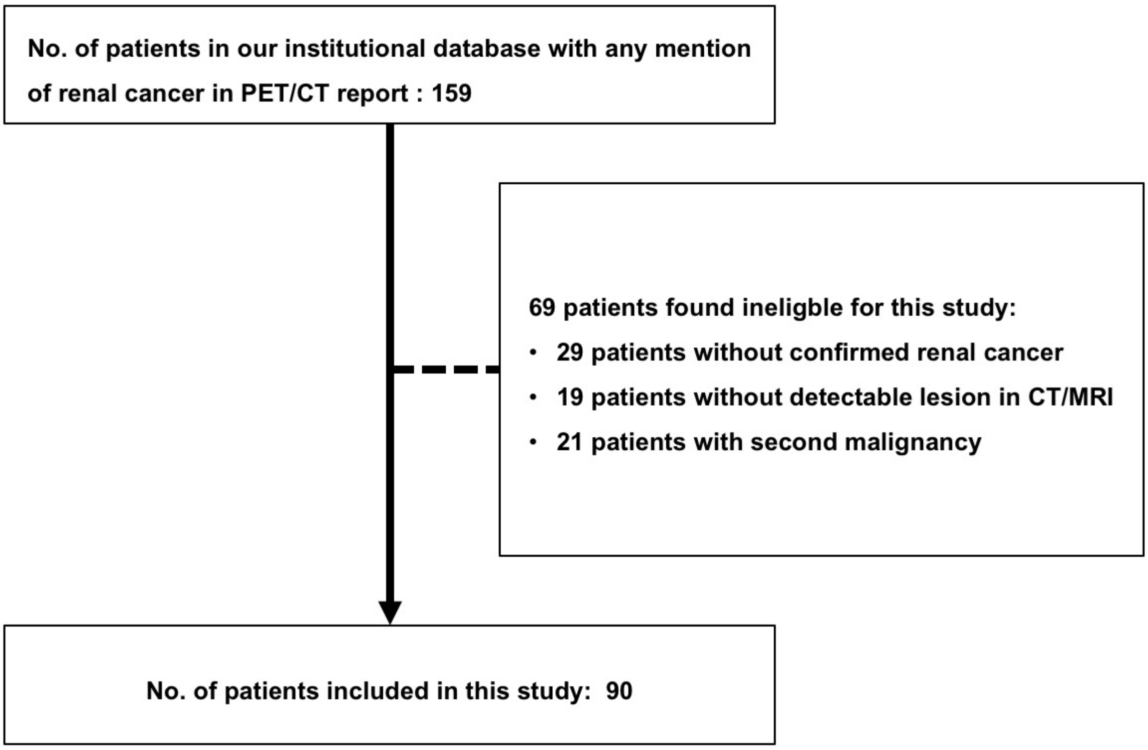

© 2022 Ferdinandus J, et al. *JAMA Network Open.*
